# Hyperoside Induces Breast Cancer Cells Apoptosis via ROS-Mediated NF-κB Signaling Pathway

**DOI:** 10.3390/ijms21010131

**Published:** 2019-12-24

**Authors:** Jinxia Qiu, Tao Zhang, Xinying Zhu, Chao Yang, Yaxing Wang, Ning Zhou, Bingxin Ju, Tianhong Zhou, Ganzhen Deng, Changwei Qiu

**Affiliations:** 1 Department of Clinical Veterinary Medicine, College of Veterinary Medicine, Huazhong Agricultural University, Wuhan 430070, China; qjx0916@163.com (J.Q.); zt951122@163.com (T.Z.); zhuxinying1994@163.com (X.Z.); chaoyang_nick@163.com (C.Y.); zhouning@webmail.hzau.edu.cn (N.Z.); jubingxin@126.com (B.J.); 18438615327@163.com (T.Z.); ganzhendeng@sohu.com (G.D.); 2 College of Veterinary Medicine, Huazhong Agricultural University, Wuhan 430070, China; wyx20132018@163.com

**Keywords:** hyperoside, apoptosis, breast cancer, ROS, nuclear transcription factor-κB (NF-κB)

## Abstract

Hyperoside (quercetin 3-*o*-β-d-galactopyranoside) is one of the flavonoid glycosides with anti-inflammatory, antidepressant, and anti-cancer effects. But it remains unknown whether it had effects on breast cancer. Here, different concentrations of hyperoside were used to explore its therapeutic potential in both breast cancer cells and subcutaneous homotransplant mouse model. CCK-8 and wound healing assays showed that the viability and migration capability of Michigan Cancer Foundation-7 (MCF-7) and 4T1 cells were inhibited by hyperoside, while the apoptosis of cells were increased. Real-time quantitative PCR (qRT-PCR) and western blot analysis were used to detect mRNA and the protein level, respectively, which showed decreased levels of B cell lymphoma-2 (Bcl-2) and X-linked inhibitor of apoptosis (XIAP), and increased levels of Bax and cleaved caspase-3. After exploration of the potential mechanism, we found that reactive oxygen species (ROS) production was reduced by the administration of hyperoside, which subsequently inhibited the activation of NF-κB signaling pathway. Tumor volume was significantly decreased in subcutaneous homotransplant mouse model in hyperoside-treated group, which was consistent with our study in vitro. These results indicated that hyperoside acted as an anticancer drug through ROS-related apoptosis and its mechanism included activation of the Bax–caspase-3 axis and the inhibition of the NF-κB signaling pathway.

## 1. Introduction

Breast cancer causes great concern for public health. According to a report about breast cancer published in 2018, the morbidity rate of breast cancer was almost equivalent to lung cancer (11.6%) and the mortality rate was the second among all cancers (6.6%) [1]. Breast cancer is the leading cause for female death, followed by colorectal cancer and lung cancer [2]. Traditional therapies for breast cancer involve surgery, radiotherapy and chemotherapy [3,4], while there are many new methods like endocrine therapy, biological target therapy and traditional Chinese medicine therapy [5,6]. Chinese medicine now has tremendous importance in chemotherapy as it has lesser side effects and toxicity [7], thus a great deal of studies has been devoted to identify novel drugs with potential for breast cancer treatment in recent years. Therefore, we need to explore the molecular mechanism of the anti-breast cancer drugs for better treatment.

The process of abnormal growth of cancer cells may be partly caused by abnormalities in reactive oxygen species (ROS) [8,9]. Endogenous ROS included superoxide anions, hydrogen peroxide and hydroxyl radicals, which are generated from oxidative phosphorylation in the mitochondrial inner membrane by electron transport [10]. Cell proliferation and cell survival need a certain level of ROS. Under normal circumstances, a certain level of ROS is used to maintain the balance of proliferation and apoptosis of cells. Under physiological conditions, low levels of ROS can activate tyrosine kinases. Then, the NF-κB signaling pathway and hypoxia-inducible factor are activated for cell growth. On the other hand, both excessive and an extremely low amount of ROS result in ROS-mediated signaling cascades; therefore, increasing the chances of cell death and inhibiting the cell growth [11]. Both these two approaches were used for the treatment of cancers. These two characteristics represent its two possible effects with regard to the treatment of cancers [11].

The nuclear factor-κB (NF-κB) transcription factor family has a major effect on the inflammatory process, which is the mediators of inflammatory processes and have utter importance in the innate and adaptive immune response. Activation of NF-κB happens commonly in tumors, and inflammatory cytokines will change in tumor microenvironment [12]. Activated NF-κB pathway can bind with DNA sequences, activating the expression of multiple genes. Those genes can develop cell proliferation, regulate apoptosis, enhance angiogenesis and stimulate cells’ invasion and metastasis ability [13,14]. Changing the expression of NF-κB can alter tumor cells’ proliferation and apoptosis ability [15,16]. Studies have found that lncRNAs interact with the target protein’s functional domains, which inhibits cancer metastasis by inhibiting the activation of NF-κB pathway [17]. In conclusion, drugs that can inhibit NF-κB pathway are showing a promising future for cancer treatment.

Hyperoside (quercetin 3-*o*-β-d-galactopyranoside) is one of the flavonoid glycosides with anti-inflammatory, antidepressant and anti-cancer effects [18]. The biofunctions of hyperoside were mainly involved in antioxidants, hypoglycemic, anti-cancer, anti-inflammatory and cardiovascular effects [19]. Previous studies have showed that hyperoside can help with the amelioration of lung cancer [20], pancreatic cancer [21], prostate cancer [22] and colon cancer [23]. However, whether hyperoside has therapeutic effects on breast cancer remains elusive. For the first time, our study proved the treatment efficacy of hyperoside and its mechanism in breast cancer via the ROS-mediated NF-κB signaling pathway.

## 2. Results

### 2.1. Effects of Hyperoside on Cell Viability

**Figure 1:**
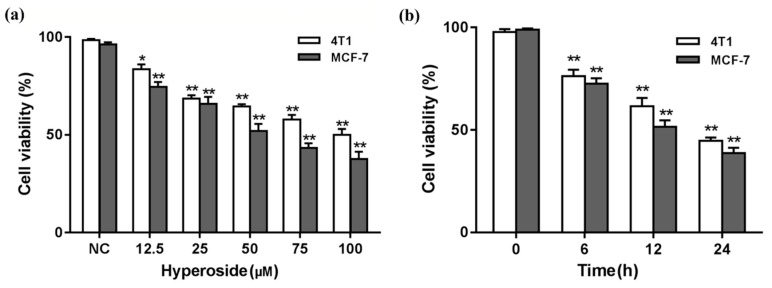
Effects of hyperoside on cell viability. A CCK-8 assay was used to determine the cytotoxicity of hyperoside on MCF-7 cells and 4T1 cells. Calculate cell viability according to the equation. (**a**) MCF-7 cells and 4T1 cells were exposed to different concentrations of hyperoside (12.5, 25, 50, 75, or 100 µM); (**b**) MCF-7 cells and 4T1 cells were exposed for different time periods (6, 12, or 24 h) of hyperoside. Data are expressed as mean ± SD of three independent experiments. * *p* < 0.05; ** *p* < 0.01 (Student’s *t*-test).

In order to avoid cells preference of drugs, we chose MCF-7 cells and 4T1 cells. Then, cytotoxicity of hyperoside on MCF-7 cells and 4T1 cells was determined by CCK-8. The results from Figure 1a,b show the changes of cell viability with different concentrations and different time periods. Considering the cell viability after the treatment of hyperoside, a time- and concentration-dependent pattern was shown.

### 2.2. Hyperoside Can Cause Apoptosis on Breast Cancer Cells

**Figure 2:**
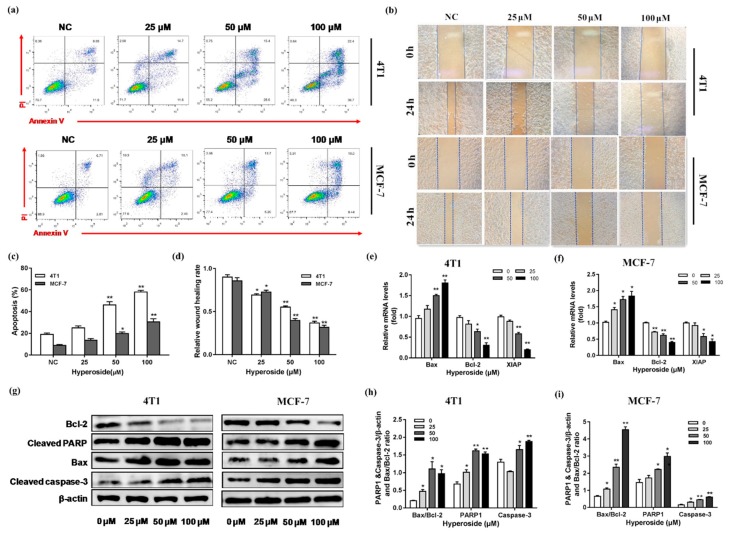
Effects of hyperoside on apoptosis and migration of breast cancer cells. MCF-7 cells and 4T1 cells were used to determine the extent of apoptosis and migration in the presence of hyperoside. (**a**,**c**) MCF-7 cells and 4T1 cells were stimulated with different concentrations (25, 50 and 100 µM) for 24 h. Then, annexin-V-FITC and propidine iodide (PI) kit was used to stain the harvested cells, of which later flow cytometry would be performed; (**b**,**d**) hyperoside (25, 50 and 100 µM) for 24 h were used to perform the wound healing assays; (**e**,**f**) qRT-PCR was used to detect the mRNA levels of Bax, Bcl-2, and XIAP. GAPDH was used as the normalization; (**g**–**i**) Western blot was used to examine the protein levels of cleaved caspase-3, Bax, cleaved PARP, and Bcl-2. β-actin was used for normalization. Data are expressed as mean ± SD of three independent experiments. * *p* < 0.05, ** *p* < 0.01 (Student’s *t*-test).

It is known that hyperoside has cytotoxicity on various cancer cells [24,25,26]. Further exploring the effects of hyperoside in these cells, we used flow cytometry and a cell scratch assay to assess the extent of apoptosis and migration induced by hyperoside. We found the apoptosis in MCF-7 cells and 4T1 cells was increased with the increased concentration of hyperoside (Figure 2a,c). Correspondingly, the wound healing assay indicated hyperoside could inhibit cell migration (Figure 2b,d). Meanwhile, the results of qRT-PCR showed that after treating hyperoside with different concentrations, the expressions of Bax, XIAP, and Bcl-2 were activated or inhibited in 4T1 and MCF-7 cells (Figure 2e,f). Under the administration of hyperoside, the expressions of Bax, cleaved caspase-3 and cleaved PARP increased and Bcl-2 decreased both in mRNA and protein levels (Figure 2g–i). These data showed that hyperoside caused apoptosis in breast cancer cells.

### 2.3. Hyperoside Induces Apoptosis by Reducing Intracellular ROS Levels

**Figure 3:**
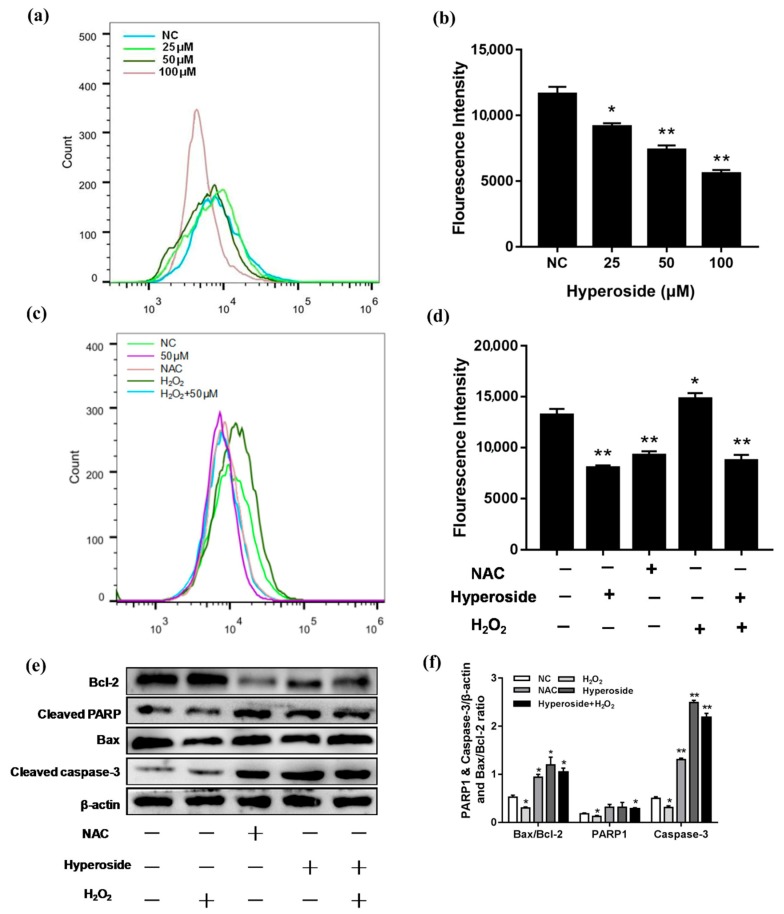
Hyperoside induces apoptosis by reducing intracellular ROS levels. (**a**–**d**) 4T1 cells were stimulated with different concentrations (25, 50 and 100 µM). NAC: Cells were stimulated with 10 mM NAC; H_2_O_2_: Cells were stimulated with 25 µM H_2_O_2_; hyperoside + H_2_O_2_: Cells were co-stimulated with 25 µM H_2_O_2_ and 50 µM hyperoside. All these groups were performed for 24 h. The intracellular ROS levels were measured by staining with DCFH-DA (10 mM) for 30 min and then determined by flow cytometry. (**e**,**f**) 4T1 cells were stimulated with hyperoside (50 µM), NAC (10 mM), H_2_O_2_ (25 µM), and hyperoside (50 µM) + H_2_O_2_ (25 µM) for 24 h, respectively. The proteins levels of cleaved caspase-3, Bax, cleaved PARP, and Bcl-2. β-actin were used for normalization. Data are expressed as mean ± SD of three independent experiments. * *p* < 0.05, ** *p* < 0.01 (Student’s *t*-test).

Hyperoside could decrease intracellular ROS levels [27]. In order to further explore the mechanism of hyperoside promoting apoptosis on breast cancer cells, we tested ROS levels after the stimulation of hyperoside. We used ROS scavenger NAC as a negative control and H_2_O_2_ as a positive control. Results showed that the ROS level was decreased with the increased concentration of hyperoside (Figure 3a,b). The hyperoside reduced the generation of ROS in 4T1, functioning the same as the NAC group (Figure 3c,d). Meanwhile, western blot detected the effects of H_2_O_2_ and reactive oxygen scavenger NAC on apoptotic protein levels in 4T1 cells. As shown in Figure 3e,f, hyperoside, NAC, and the cotreatment of hyperoside and H_2_O_2_ treatment group increased the protein levels of Bax, cleaved caspase-3, and cleaved PARP with decreased levels of Bcl-2, while the H_2_O_2_ treatment group showed the opposite results. These results indicated that reduction of ROS levels might be the reason of hyperoside-induced apoptosis.

### 2.4. Hyperoside Inhibits NF-κB Signaling Pathway via ROS

**Figure 4:**
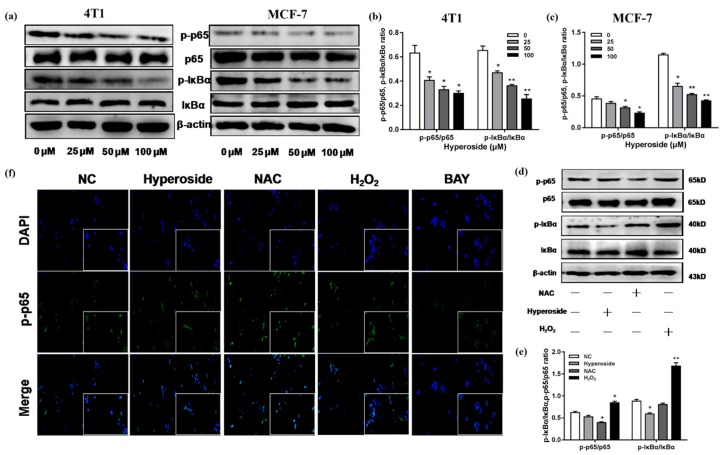
Hyperoside inhibits the NF-κB signaling pathway via ROS. MCF-7cells and 4T1 cells were stimulated with hyperoside (25, 50 and 100 µM), NAC (10 mM), H_2_O_2_ (25 µM), and hyperoside (50 µM) for 24 h, respectively. (**a**–**e**) Western blot was used to determine the proteins level of p65, p-p65, IκB-α, and p-IκB-α. β-actin was used for normalization. (**f**) Immunofluorescence images hyperoside and NAC inhibited p-p65 activation in 4T1 cells. Data are expressed as mean ± SD of three independent experiments. * *p* < 0.05, ** *p* < 0.01 (Student’s *t*-test).

The generation of intracellular ROS could inhibit the NF-κB signaling pathway [28], while whether hyperoside can promote apoptosis through the inhibition of NF-κB by the attenuation of ROS remained unknown. To further investigate the hyperoside function in NF-κB pathway, we examined the expression levels of IκBα and p65 with different hyperoside concentrations in both MCF-7 and 4T1 cells. As shown in Figure 4a–c, the phosphorylation levels of IκBα and p65 were decreased in MCF-7 cells and 4T1 cells compared to the control group, and decreased with the increase of hyperoside concentration. ROS is the second messenger which is involved in various signaling pathways, including the NF-κB signaling pathway [29]. Then, experiments were conducted to determine whether ROS reduction elicited the inhibition of the NF-κB pathway. As the results show in Figure 4d,e, the hyperoside and NAC treatment groups decreased the expression level of phosphorylated IκBα and p65, while the H_2_O_2_ treatment group showed the opposite. It was also confirmed by the immunofluorescence (Figure 4f). All these statistics demonstrated that hyperoside inhibited the activation of the NF-κB signaling pathway via the attenuation of intracellular ROS generation.

### 2.5. Hyperoside Can Inhibit the Growth of Breast Tumor

**Figure 5:**
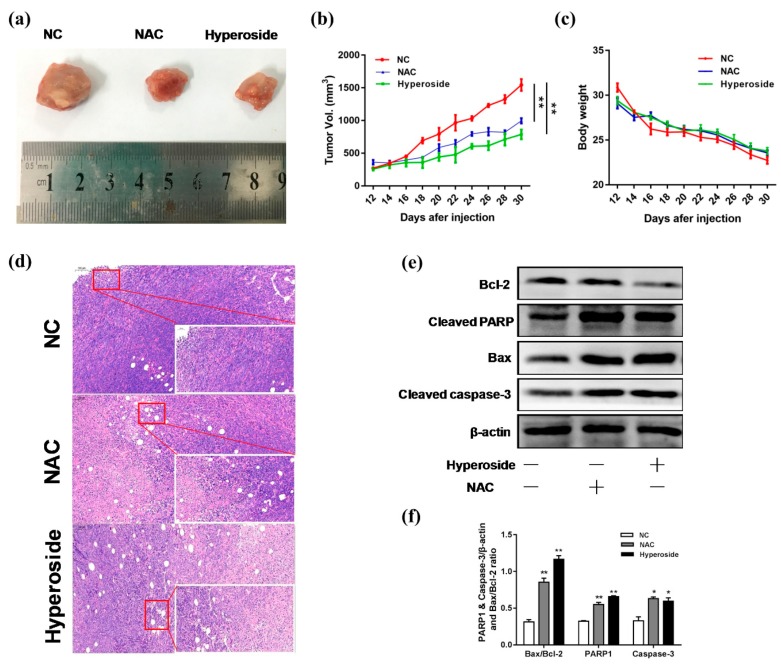
Effects of hyperoside on tumor growth. Balb/c mice were divided into three groups (normal control (NC) group, hyperoside group, and NAC group). (**a**–**c**) Day 0 was the day subcutaneous homotransplant mouse model was created. On Day 12, drugs were injected into the tumor. The volume of the tumor and mouse weights were measured every two days (*n* = 5 per group). On Day 30, the mice were killed, and samples were collected. (**d**) H&E staining was performed. (**e**,**f**) Western blot was used to determine the protein expression of cleaved caspase-3, cleaved PARP, Bax, and Bcl-2. β-actin was used for normalization. Data are expressed as mean ± SD of three independent experiments. * *p* < 0.05, ** *p* < 0.01 (Student’s *t*-test).

To evaluate the effect of hyperoside on tumor growth in vivo, we used a subcutaneous homotransplant mouse model. As shown in Figure 5a–c, compared to the control group, the average tumor volume in the hyperoside-treated group was significantly reduced, which was also confirmed by H&E staining of tumor sections in each treatment group (Figure 5d). We further examined the expression of Bax and cleaved caspase-3. As shown in Figure 5e,f, Bcl-2 was decreased in the hyperoside-treated group, while Bax and cleaved caspase-3 were on the rise. All these results showed that apoptosis was induced in vivo when the tumors were injected with hyperoside, which resulted in the reduction of tumor volume.

## 3. Discussion

**Figure 6:**
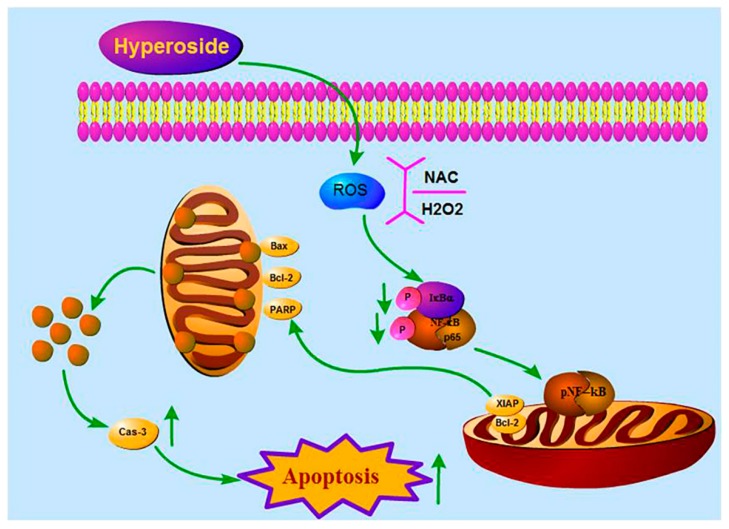
Inhibition of hypericin on ROS-mediated NF-κB signaling and target gene expression in 4T1 cells. Hyperoside inhibited the activation of the NF-κB pathway and reduced translocation of p-p65 in the nucleus by reducing ROS production in 4T1 cells. Inhibition of NF-κB down-regulates the transcription of anti-apoptotic genes, such as Bcl-2 and XIAP, but it also induced the accumulation of Bax, which leads to mitochondrial dysfunction and leads to apoptosis by activating caspase-3.

Breast cancer is one of the main malignant tumors in women which became a global treatment problem [1]. Although chemotherapy is still prevalent in cancer treatment, the characteristics of breast cancer treatment resistance, high rate metastasis, and poor prognosis are plagued by scientists for a long time [30]. Traditional Chinese medicine is considered to be a new potential therapeutic agent with more tolerance [31]. Hyperoside is one of the main active compounds in the leaves of *Zanthoxylum bungeanum* (*Z. bungeanum*). Previous studies confirmed that hyperoside induced apoptosis in various cancer cell lines [25,26,32], but how hyperoside induced apoptosis in breast cancer cells was unknown. In our study, we demonstrated that hyperoside can induce apoptosis and inhibit proliferation both in vitro and in vivo, which further clarified the mechanism leading to such cell killing. We found that hyperoside could deactivate the NF-κB pathway and reduced intracellular ROS levels, resulting in lowered anti-apoptotic genes’ expression (XIAP and Bcl-2) and the accumulation of Bax. Since the NF-κB pathway target gene can inhibit the activation of caspase-3, hyperoside indirectly inhibited the NF-κB pathway target gene expression, induced mitochondrial dysfunction, and activated caspase-3 to induce breast tumor’s apoptosis, as we summarized in Figure 6.

Previous studies have shown that hyperoside was vital in inhibiting the proliferation and migration of lung cancer cells [24,26]. The growth and metastasis characteristics of 4T1 cells in BALB/c mice are very similar to human breast cancer, so we chose the 4T1 mouse mammary cancer cell line. Our results showed that proliferation rates of MCF-7 cells and 4T1 cells were prohibited in a time- and concentration-dependent manner. The effect of hyperoside on cell proliferation also affected cell migration, as demonstrated by the fact that wound healing rates decreased with increasing concentrations. In addition, flow cytometry showed that the apoptosis of MCF-7 and 4T1 cells increased while hyperoside concentrations were higher, which was consistent with previous studies [26]. Furthermore, results from western blot showed that, compared with the control group, Bax, cleaved caspase-3, and cleaved PARP were significantly increased and Bcl-2 was decreased in MCF-7 cells and 4T1 cells. Previous studies have shown that the Bcl-2 family can inhibit mitochondria-induced apoptosis [33], and we found that the Bax:Bcl-2 ratio increased in the hyperoside group, suggesting that hyperoside was likely to induce apoptosis of breast cancer cells through endogenous pathways.

It has been reported that almost all cancer cases are accompanied by the increase of ROS. Due to the increase of ROS, the cancer-related signal transduction pathway is stimulated to enhance the survival and proliferation of cancer cells [34]. ROS destroy the cellular homeostasis regardless of whether the ROS level rises or falls [35,36]. Hyperoside reduces intracellular ROS, mainly through the induction of HO-1, to protect cells from oxidative stress [27]. This hypothesis was then confirmed that hyperoside regulates oxidative damage in *Saccharomyces cerevisiae* by reducing ROS [37]. Hyperoside reduced the impact of H_2_O_2_ oxidation in human umbilical vein endothelial cells via ERK signaling where ROS suffered a reduction [38]. Hyperoside reduced intracellular ROS to regulate mitochondrial apoptotic pathways and prevent oxidative damage [39,40]. In our study, hyperoside reduced the intracellular ROS level, which implied that hyperoside may regulate the mitochondrial apoptotic pathway and prevent oxidative damage. Meanwhile, Western blot analysis showed that, both in the hyperoside treatment group and the NAC treatment group, Bax/caspase-3/PARP-1 increased while Bcl-2 was reducing. This result indicated that hyperoside did induce apoptosis by affecting the mitochondrial pathway in 4T1 cells.

ROS interacts with NF-κB signaling. The level of NF-κB activity is also regulated by ROS levels, which can be activated or inhibited [29]. There are articles illustrating that the inhibition of the NF-κB signaling pathway could induce breast cancer cells’ apoptosis [41]. NF-κB regulates apoptosis by inducing the expression of several anti-apoptotic genes, including the XIAP and Bcl-2 families [42,43]. In our experiment, we found hyperoside reduced intracellular ROS levels, while inhibiting Bcl-2 and XIAP expression. Therefore, we speculated that hyperoside-induced apoptosis might be related to the NF-κB signaling pathway. Hyperoside reduced neurotoxicity of microglial cells by inhibiting the phosphorylation of p38 and p65 proteins [26], and it could inhibit tumor necrosis factor-alpha-mediated vascular inflammation [44]. We evaluated proteins of NF-κB pathway in MCF-7 cells and 4T1 cells. We found that the hyperoside treatment group inhibited phosphorylation of p65 and IKBα, while the action of H_2_O_2_ altered the opposite. In addition, the decrease of ROS may also cause mitochondrial dysfunction and lead to apoptosis by activating caspase-3. We also confirmed this by in vivo experiments, which was manifested by reduction of tumor volume. That anti-apoptotic protein decreased while pro-apoptotic protein increased, which was found in the western blot analysis of tumor tissues.

In general, our experiments indicated that hyperoside could deactivate NF-κB signaling pathway by firstly reducing intracellular ROS levels, thereby promoting apoptosis in breast cancer cells. 

## 4. Materials and Methods

### 4.1. Cell Culture

The 4T1 cells were provided by the Chinese Academy of Sciences Cell Bank (Shanghai, China), and the MCF-7 cells were supplied by the American Type Culture Collection (ATCC, Manassas, VA, USA). The cells were grown in RPMI 1640 medium with 10% fetal bovine serum (Gibco-Life Technologies, Carlsbad, CA, USA) and 1% penicillin/streptomycin (Gibco-Life Technologies, Carlsbad, CA, USA) in T25 cell culture flasks.

### 4.2. Reagents and Antibodies

**Figure 7:**
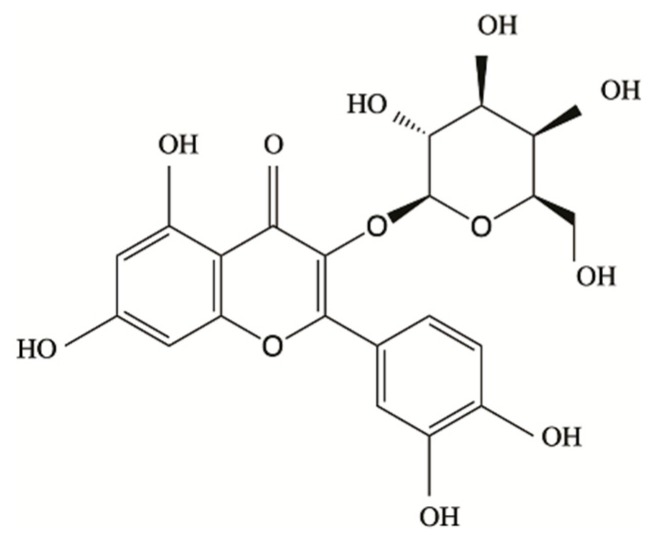
Chemical structure of hyperoside.

Hyperoside (C_21_H_20_O_12_, purity ≥ 99%, relative molecular mass = 464.38) was from Despite Biotech (Chengdu, China) (Figure 7). Primary antibodies for β-actin (#3700), anti-p-NF-κB p65 (#3033), anti-NF-κB p65 (#8242), anti-p-IκBα (#2859), and anti-IκBα (#9242) were purchased from Cell Signaling Technology (Beverly, MA, USA). Anti-cleaved caspase-3 (#ab184787) and anti-cleaved PARP (#ab32064) were obtained from Abcam (Cambridge, UK). Anti-Bax (#sc-493) and anti-Bcl-2 (#sc-492) were obtained from Santa Cruz Biotechnology (Santa Cruz, CA, USA). In addition, we bought the N-acetyl-cysteine (NAC) from Sigma-Aldrich Chemical (Shanghai, China). IκB-α inhibitor BAY11-7082 and annexin V-FITC Apoptosis Detection Kit were purchased from the Beyotime Institute Biotechnology (Shanghai, China).

### 4.3. Cell Viability Assay

The extent of hyperoside’s cytotoxicity on MCF-7 cells and 4T1 cells was examined by the Cell Counting Kit-8 (Tokyo, Japan). There were five repeats for one group, and when density reached 5 × 10^3^ cells/mL (37 °C, 12 h), they were added with hyperoside (50 μM) according to different time periods (6, 12, or 24 h) and the normal control group, and different concentrations of hyperoside (12.5, 25, 50, 75, or 100 μM) for 24 h. A total of 10 μL (5 mg/mL) CCK-8 was added for 2.5 h. Optical density (OD) was read on a microplate reader at an absorbance value of 450 nm. Each experiment was repeated three times. Data was expressed as mean ± SD.

(1) $$\mathrm{Cell}\;\mathrm{viability}=\frac{OD_{Treatment\;Group}-OD_{Blank\;Group}}{OD_{Control\;Group}-OD_{Blank\;Group}}$$

### 4.4. Intracellular ROS Assay

When reaching 1 × 10^6^ cells/mL, cells were divided into 6-well plates for 12 h. The indicated concentrations of hyperoside were added into cell plates for 24 h. After the treatment, as described before, cells were washed with pre-chilled phosphate buffer saline (PBS) and stained with cell-permeable 2′, 7′-dichlorofluorescein diacetate (DCFH-DA, Beyotime) at 37 °C for 30 min in the dark, and then washed by PBS to eliminate extracellular DCFH-DA to fluorescent dichlorofluorescein (DCF). Flow cytometry (Becton Dickinson; Franklin Lakes, NJ, USA) was then used to obtain the data.

### 4.5. Apoptosis Assay

Annexin V-FITC (Fluorescein isothiocyanate) and propidium iodide (PI) double staining were used to perform the apoptosis trials. MCF-7 cells and 4T1 cells were used, they were first cultured in 6-well plates for 12 h. Then different concentrations of hyperoside (25, 50, or 100 μM) were added for 24 h. They were centrifuged at a speed of 1200 r/min for 8 min, washed three times with cold PBS, and resuspended in binding buffer (400 μL). The cells were then incubated with Annexin V-FITC (5 μL) and PI (5 μL) for 20 min at 25 °C, shaded from light. Cell sorting analysis of collected cells, adherent cells, and flowing cells were performed by flow cytometry (Becton Dickinson; Franklin Lakes, New Jersey).

### 4.6. Wound-Healing Migration Assay

As the previous study described [24], MCF-7 cells and 4T1 cells were placed onto 6-well plates. When cells grew to 80% confluence, the cell monolayer was scraped by sterile 200 μL plastic pipette tips, and then cells were washed twice. After washing, fresh medium containing various concentrations of hyperoside was added at different time periods (6, 12, or 24 h). Serum-reduced Opti-MEM I medium were obtained from Invitrogen, Carlsbad, CA, USA. Images of wound closure were recorded by an inverted microscope.

### 4.7. Quantitative Polymerase Chain Reaction Assay

TRIzol reagent (Invitrogen, Carlsbad, CA, USA) was used to obtain total RNA and reverse transcription assay was performed to acquire cDNA and the kit was from Takara, Japan. The 2×SYBR green-master mix (Roche Diagnostics, Mannheim, Germany) was used and the LightCycler 96 instrument (Roche Diagnostics, Germany) was the instrument used to obtain data. The reference gene was GAPDH according to standard protocols with three repeats. The primer sequences are shown in Table 1. The 2^−ΔΔCt^ comparative method was the formula used for the calculation of target genes.

### 4.8. Western Blot Analysis

Cells were treated with previous concentrations of hyperoside, NAC, and H_2_O_2_ peroxide for 24 h, then collected after being washed three times with PBS. A mix, which included radio immunoprecipitation assay (RIPA), phosphatase inhibitors, and PMSF, was used to extract the total protein (BioSharp, Hefei, China). The total protein concentrations were determined by the Pierce BCA Protein Assay Kit (Thermo Fisher Scientific, Rockford, IL, USA). A 10% sodium dodecyl sulfate (SDS)-polyacrylamide gel was used for electrophoresis, then electric transfer was conducted with a polyvinylidene difluoride (PVDF) membrane for 2 h at 120 V. A 5% skim milk was used for membrane blocking for 3 h. Primary antibodies were incubated overnight with the indicated proteins (1:1000), stored at 4 °C; then, the membrane was washed three times with Tris-buffered saline tween (TBST) for 10 min. Secondary antibody (1:5000) was used for 2 h at 25 °C. Finally, the Vilber Lourmat Fusion FX7 Detection System (France) was used for the acquirement of protein images. β-actin served as the internal standard.

### 4.9. Immunofluorescence Staining

When reaching a density of 1 × 10^5^ cells/mL, 4T1 cells were planted onto a 6-well plate with slides. Immunofluorescence staining was performed after hyperoside was added as indicated for 24 h. Cells were fixed by 4% paraformaldehyde and then washed three times with PBS. Subsequently, the cells were blocked with goat serum for 30 min and incubated with primary antibodies overnight with a temperature of 4 °C, and then treated with the fluorescein (FITC)-conjugated AffiniPure Donkey Anti-rabbit IgG (H+L) in the dark for 2 h. The proteins were detected with 4,6-diamidino-2-phenylindole (DAPI; Beyotime) for nuclear counterstaining. A laser scanning confocal microscope (Zeiss Germany, Oberkochen, Germany) was used for the acquirement of images.

### 4.10. In Vivo Experiment

Balb/c mice at 8–10 weeks old (25–30 g) were purchased from the Hubei Province Experimental Animal Center of Huazhong Agricultural University (Wuhan, China). Institutional Ethical Committee for Animal Care and Use of Huazhong Agricultural University (HZAUMO-2015-12) and United States National Institutes of Health were the guidelines for experimental animal care and use.

The mice were reared for a week after we carried out the experiment. Approximately 1.0 × 10^7^ 4T1 cells were harvested and suspended in 100μL PBS. Then we injected those cells into the fourth breast pad of the mice. Mice were randomized into three groups after rearing for 12 days, and the following administrations were executed: Hyperoside (50 mg/kg i.p. every two day for 18 days), NAC (100 mg/kg i.p. every two day for 18 days), or saline as the control group. Bodyweight and tumor volume were measured every two days. Tumor volume (V) = 0.5 × length × width^2^. Mice were then sacrificed after we finished the administration, and 4% paraformaldehyde was used for H&E.

### 4.11. Histopathological Assessment

Tumor tissues of the mice were harvested and we used a section of 1 cm in size for further research. Tissues were fixed in 10% formalin. The tissues were dehydrated then embedded by paraffin, which were later cut into 5 μm thick slices for hematoxylin and eosin (H&E) staining. An optical microscope (Olympus, Japan) was used for the acquirement of images.

### 4.12. Statistical Analysis

Data are presented as means ± SD with at least three independent experiments. The significance was calculated by one-way ANOVA or Student’s t-test (GraphPad Prism 7). *p* < 0.05 was considered statistically significant and *p* < 0.01 was extremely significant (* *p* < 0.05, ** *p* < 0.01).

## 5. Conclusions

In summary, our experiments show that hyperoside can act as an anticancer drug by inhibiting NF-κB signaling and activating the Bax-caspase-3 axis through ROS-induced apoptosis. These data indicated that hyperoside has great potential as an anti-breast cancer drug and deserved further study in the future.

## Figures and Tables

**Table 1:** Primer sequence for qPCR.

Gene	Sequence (5′-3′): Forward and Reverse	GenBank Accession Numbers
GAPDH	CAGCTACTCGCGGCTTTAC CCCTGCTTATCCAGTCCTAGC	NM_008084.3
Bax	CTGGATCCAAGACCAGGGTG CCTTTCCCCTTCCCCCATTC	NM_007527.3
Bcl-2	TCTTTGAGTTCGGTGGGGTC AGTTCCACAAAGGCATCCCAG	NM_009741.5
XIAP	CTGGCCGGACTATGCTCATT CACGATCACAGGGTTCCCAA	NM_001301639.1

## Abbreviations

Bcl-2	B cell lymphoma-2
MCF-7	Michigan Cancer Foundation-7
PI	Propidine iodide
CCK-8	Cell Counting Kit-8
ROS	Reactive oxygen species
NAC	N-acetyl-l-cysteine
XIAP	X-linked inhibitor of apoptosis
qRT-PCR	quantitative real-time PCR
NF-κB	Nuclear transcription factor-κB
NC	normal control
ERK	extracellular regulated protein kinase
*Z. bungeanum*	*Zanthoxylum bungeanum*
